# Research on Alzheimer’s disease MRI image classification based on spatial attention mechanism

**DOI:** 10.3389/fnagi.2026.1657578

**Published:** 2026-04-10

**Authors:** Shanshan Zhao, Haolin Shi, Changhao Guan, Qingjia Zeng, Chongyang Zhang, Yanli Wan

**Affiliations:** Institute of Medical Information/Library, Chinese Academy of Medical Sciences and Peking Union Medical College, Beijing, China

**Keywords:** Alzheimer’s disease (AD), deep learning, magnetic resonance imaging (MRI), medical image classification, spatial attention mechanism, Swin Transformer

## Abstract

**Introduction:**

Early diagnosis of Alzheimer’s Disease (AD) is crucial for improving patient quality of life and treatment outcomes. However, accurately classifying MRI scans of AD remains challenging due to the subtle and spatially complex nature of lesion regions. This study proposes a novel bidirectional spatial attention mechanism to enhance the focus on key pathological features in AD MRI images, aiming to improve classification accuracy and support earlier intervention.

**Methods:**

To enhance model performance, we introduced a customized bidirectional spatial attention module (ATT) integrated into a Swin-Tiny Transformer backbone. Unlike conventional attention methods, the ATT module generates spatial attention maps by adaptively pooling features along both vertical and horizontal orientations, allowing refined adjustment of attention weights across different image regions. Furthermore, to address issues of limited sample size and class imbalance, we employed data augmentation and expansion strategies, enriching the diversity of training data. The model was trained and evaluated on the augmented OASIS1 dataset.

**Results:**

The improved Swin-Tiny+ATT model demonstrated significant performance gains across all key metrics on the augmented dataset. Compared to the baseline Swin Transformer, accuracy improved from 84.83% to 87.96%, recall from 89.82% to 91.92%, precision from 85.27% to 91.98%, and the F1 score from 87.26% to 91.89%. These results confirm that the ATT module effectively enhances the model’s ability to capture complex spatial features and identify critical lesion regions.

**Discussion:**

The proposed Swin-Tiny+ATT model exhibits strong potential for improving MRI-based classification of Alzheimer’s Disease. The bidirectional spatial attention mechanism successfully directs the model’s focus to relevant anatomical regions, contributing to higher precision and recall. Combined with data augmentation strategies, the approach mitigates class imbalance and enhances generalization. This work provides a promising deep learning framework to support early and accurate diagnosis of AD, with implications for clinical decision-making and personalized treatment planning.

## Introduction

Alzheimer’s disease (AD) is the most prevalent form of dementia among the elderly, accounting for approximately 60 to 80% of all dementia cases (Filiou et al., 2020). AD has a profound impact on patients’ lives and imposes a significant economic burden on their families. Mild Cognitive Impairment (MCI) serves as a precursor stage for dementia and neurodegenerative diseases, representing a critical period for potential intervention (Rosenberg and Lyketsos, 2008). Therefore, the identification of MCI and effective intervention during this variable window can help delay or even prevent the onset of Alzheimer’s disease, which has become an important objective.

In the diagnostic process of AD, medical imaging technologies have become one of the most crucial tools for physicians in diagnosis and decision-making. By utilizing MRI images to extract structural features of the brain, it is possible to detect changes in these areas and identify the need for timely treatment. Medical imaging can assist healthcare professionals in diagnosing and treating AD more accurately. However, the application of medical images often requires substantial labeling efforts, which significantly increases the workload for medical personnel. To address the issues of time consumption, subjectivity, and reliance on the physician’s experience and capabilities in the analysis of medical images, computer-aided methods for AD image analysis, particularly for MRI images have emerged as an important research direction.

Medical image classification is a key area within medical image analysis, involving the prediction of one or more labels corresponding to a query image based on its assigned categories. This process holds significant research importance within the field of computer vision. Deep learning-based medical image classification plays a crucial role in assisting medical diagnostics, expediting image reading, reducing patient waiting times, and alleviating the workload of radiologists. The process of medical image classification is consistent with general image recognition workflows, comprising three main steps: feature extraction from the input image, selection of important features, and conversion of feature information into output label categories.

Current mainstream medical image classification models based on Convolutional Neural Networks (CNNs) exhibit challenges such as low classification accuracy and insufficient generalization. It is difficult to identify a model suitable for diverse medical image classification tasks. Furthermore, due to the difficulties associated with medical image acquisition and the high costs of manual annotation, labeled medical image data samples are scarce, limiting the effectiveness of conventional deep learning models in achieving accurate classification. Additionally, CNNs lack the ability to model long-range relationships effectively (Chen et al., 2021). However, in clinical practice, these challenges are further exacerbated by the issue of class imbalance and data scarcity (Ghosh et al., 2024). Class imbalance, where certain disease stages are underrepresented in datasets, can lead to biased model predictions, resulting in reduced diagnostic accuracy and limited model generalizability in real-world scenarios. Similarly, the scarcity of labeled data, especially for rare disease stages, directly impacts the model’s ability to generalize across different patient populations, making it harder to deploy these models in clinical settings where diverse patient data is encountered. These challenges pose significant barriers to the reliable application of deep learning-based medical image classification in diagnosing Alzheimer’s disease.

In contrast, deep self-attention networks (Transformers) (Vaswani et al., 2017) possess a natural aptitude for modeling global features and long-range dependencies, which are crucial for distinguishing subtle variations in medical images, such as detecting small lesions or differentiating between closely related stages of a disease. This capability makes Transformers particularly effective in medical image analysis, where spatial context and intricate details across the entire image are critical for accurate diagnosis. Complementing the inductive bias characteristics of CNNs. Inspired by their substantial success in natural language processing, Transformers have been widely applied to various tasks in computer vision, particularly in medical image analysis, demonstrating commendable performance in applications such as object detection (Carion et al., 2020), semantic segmentation (Wang et al., 2021), object tracking (Ma et al., 2022), image generation (Jiang et al., 2021), and image enhancement (Chen et al., 2021).

This study is based on the Swin Transformer model, with improvements made to the network structure to enhance the classification and recognition of three types of MRI images related to Alzheimer’s disease: NonDemented, MildDemented, and ModerateDemented. The main contributions of this study are as follows: (1) An innovative bidirectional spatial attention mechanism is introduced in this work to strengthen spatial feature representation, leading to notable improvements in model performance across various tasks. (2) A Swin-Tiny+ATT Alzheimer’s disease classification model is proposed by incorporating a spatial attention mechanism and Swin Transformer, improving the model’s ability to capture complex spatial information and enhancing classification accuracy. (3) Various data augmentation techniques were applied to expand the training data and increase its diversity, and the impact of data augmentation on improving model performance was explored.

## Related work

The Transformer model, introduced by Google in 2017, is a seminal model in Natural Language Processing (NLP) (Vaswani et al., 2017). Dosovitskiy et al. (2020) proposed the Vision Transformer (ViT) model, which was the first to apply the original Transformer architecture to image classification tasks. Swin Transformer, introduced by Chinese researchers at Microsoft Research in 2021, is a novel Transformer model for computer vision (CV) tasks, succeeding ViT (Liu et al., 2021). Swin Transformer adopts a convolutional localization approach and builds its network using self-attention mechanisms. By leveraging self-attention to establish global correlations, it is able to capture long-range dependencies in images, making it particularly effective in handling images with complex semantic structures. Unlike ViT, which computes correlations between all tokens, Swin Transformer employs a sliding window mechanism that focuses on neighboring tokens (four or five) for correlation, gradually expanding the receptive field. This approach reduces the number of parameters, significantly decreases computational complexity, and improves model efficiency. Additionally, Swin Transformer introduces inter-layer connection mechanisms that facilitate information flow and feature extraction, further enhancing model performance. Therefore, this study selects the Swin Transformer model as the foundational model for Alzheimer’s disease MR image classification research.

In the field of AD classification by Transformer model, various innovative approaches have been employed, leveraging Model Innovation, transfer learning, and multimodal data to enhance model performance and address challenges such as limited annotated data.

### Model innovations and hybrid models

Mora-Rubio et al. (2023) combined CNNs with Vision Transformers to classify AD stages from MRI scans, achieving up to 89% accuracy. Huang and Li (2023) developed the Resizer Swin Transformer (RST) to extract multi-scale features from minimally processed sMRI images. Kim et al. (2024) proposed a domain-aware multi-task learning approach to pretrain a 3D Swin Transformer using brain MRI, outperforming existing methods by incorporating domain knowledge and contrastive learning. Chen et al. (2024) introduced LongFormer, a hybrid 3D CNN and transformer designed to learn from longitudinal MRI data, addressing missing data challenges. Studies such as MaxViT (Pacal, 2024) and MetaFormer (Pacal, 2024; Ozdemir and Pacal, 2025) have shown that combining CNN modules with self-attention mechanisms can improve accuracy while reducing the number of parameters.

### Attention mechanisms for feature extraction

Attention mechanisms are crucial for feature extraction in medical image classification. Both the ConvNeXtV2 module and focal self-attention mechanisms have shown effectiveness in this area. ConvNeXtV2 improves feature extraction by capturing fine-grained details, while focal self-attention enhances the model’s ability to focus on relevant information and suppress noise, boosting performance in tasks like medical image classification (Ozdemir and Pacal, 2025). Yao et al. (2019) proposed a deep learning model for breast biopsy image classification using a perceptron-based attention mechanism, enabling dynamic weight allocation between feature pairs during training. Hybrid attention mechanisms, combining spatial and channel attention, have proven effective in medical image analysis, improving classification accuracy. Sun et al. (2019) applied such mechanisms in CNNs for endometrial adenocarcinoma diagnosis, achieving strong results. Alenezi et al. (2024) integrated EfficientNetB7 with various attention modules to enhance multi-scale recognition of retinal diseases in OCT images. Qin et al. (2021) used a 3D CNN with hybrid attention mechanisms to improve diagnostic accuracy. Tabatabaei et al. (2023) developed a dual-branch model combining self-attention and CNNs for brain tumor classification in MRI images, which aids in early Alzheimer’s disease diagnosis. These approaches significantly enhance diagnostic performance in medical imaging tasks.

The incorporation of focal self-attention mechanisms enables precise capture of fine-grained features and effective suppression of irrelevant information, thus improving performance in medical image classification. Moreover, hybrid attention mechanisms, by combining spatial and channel modules, further enhance the extraction of salient regional features, offering more efficient and accurate technical support for early diagnosis.

### Transfer learning and cross-domain techniques

Mora-Rubio et al. (2023) proposed a method leveraging knowledge from the Transfer learning and cross-domain techniques have played a vital role in the diagnosis of Alzheimer’s disease, particularly in addressing challenges related to data scarcity and model efficiency. Mora-Rubio et al. (2023) proposed a transfer learning approach that leverages knowledge from the natural image domain to alleviate the scarcity of brain imaging data. By utilizing pretrained models from natural image datasets, their method successfully transferred knowledge to the medical imaging domain and achieved performance comparable to state-of-the-art methods. This approach effectively mitigates the difficulties associated with annotating medical images and improves the model’s generalization ability. Additionally, Sarraf et al. (2023) introduced an optimized vision Transformer architecture (OViTAD), which achieved remarkable F1 scores in classification tasks. Compared to standard Transformer models, OViTAD significantly reduces the number of parameters while maintaining high performance, demonstrating the advantages of architectural optimization in enhancing computational efficiency and reducing resource consumption. This optimization also offers new directions for transfer learning, where knowledge transfer and model refinement across domains not only improve model performance in medical imaging applications but also enable more efficient resource utilization. Swati et al. (2019) proposed a CNN-based multi-class classification method using transfer learning, where a block-wise fine-tuning strategy was employed to enhance brain tumor classification accuracy on MRI images. Experimental results showed that their approach achieved an average accuracy of 94.82% on the CE-MRI dataset, outperforming traditional machine learning and other deep learning methods. Koul et al. (2024) presented a hybrid deep transfer learning model for predicting and classifying various respiratory diseases. Their model achieved a classification accuracy of 99.77% on a lung image dataset, with outstanding performance in terms of accuracy, precision, recall, and F1 score.

These studies collectively demonstrate that the integration of transfer learning and cross-domain techniques offers promising new directions for efficient medical image analysis and accurate diagnosis. Such advancements are instrumental in driving the progress of early Alzheimer’s disease detection technologies.

While Transformer models, such as Vision Transformer (ViT) and Swin Transformer, have shown promise in Alzheimer’s disease (AD) MRI classification, challenges remain in model efficiency, feature extraction, and capturing complex spatial dependencies. To address these, we introduce a novel bidirectional spatial attention module (ATT) integrated with Swin Transformer, enhancing its ability to capture both local and global spatial features. Additionally, we tackle data scarcity and class imbalance with data augmentation strategies, improving model performance and generalization. Our approach advances AD classification by improving accuracy, robustness, and efficiency in medical image analysis.

Based on existing research, this study proposes a novel Swin-Tiny+ATT architecture. Compared to existing methods: (1) Unlike the MaxViT framework used in cervical cancer diagnosis, we retain the original Swin blocks and incorporate bidirectional attention to maintain structural integrity; (2) Compared to the focal self-attention mechanism in MetaFormer, our spatial attention mechanism specifically targets hippocampal atrophy patterns; (3) Compared to the augmentation strategies used in cervical cancer research, our optimized augmentation strategy places greater emphasis on addressing data scarcity and class imbalance issues.

## Methods

### Feature extraction requirements for AD MRI images

When applying deep learning techniques for the classification of Alzheimer’s disease (AD) using MRI images, analyzing and extracting relevant features is a crucial step. Effective feature extraction from AD MRI images must meet the following requirements:

(1) Sensitivity to Structural Changes at Both Local and Global Scales: Structural alterations in AD are often localized—for example, hippocampal atrophy and cortical thinning (Aosi, 2024). Therefore, models must possess strong capabilities in capturing fine-grained local features to detect these degenerative changes. However, AD also involves more global structural changes, such as cortical atrophy and ventricular enlargement. Consequently, models should be capable of capturing long-range dependencies and associations between spatially distant regions.
(2) Ability to Extract Irregular Edge Features: AD-related features in MRI images often manifest with irregular boundaries. For instance, the margins of atrophic hippocampal regions can be highly irregular. Feature extractors must effectively capture these non-uniform patterns.
(3) Noise and Artifact Robustness: MRI images frequently contain noise and artifacts that can compromise the accuracy of feature extraction. Deep learning models are therefore required to have strong noise-robust capabilities to maintain reliable performance under such conditions.
(4) Handling Data Imbalance: In AD classification tasks, the number of normal brain samples often exceeds that of diseased samples, resulting in class imbalance. Models should incorporate strategies to mitigate this imbalance, ensuring fair and accurate learning across categories.

To address these requirements, spatial attention mechanisms offer a promising solution by adaptively adjusting the weights assigned to different regions during feature extraction. This enables the model to more accurately focus on key pathological areas and improve classification performance. The advantages of spatial attention mechanisms are reflected in several key aspects:

(1) Enhanced Representation of Critical Pathological Regions: AD predominantly affects specific brain areas such as the hippocampus, amygdala, and cortical regions (Aosi, 2024). These affected regions are often small and may be obscured by background information in MRI scans. Spatial attention mechanisms dynamically assign higher weights to these crucial areas, allowing the model to concentrate on pathological features during feature extraction and thereby improving classification accuracy.
(2) Integration of Global and Local Information: AD-related abnormalities are not confined to isolated regions but involve structural changes across multiple brain areas. Spatial attention mechanisms facilitate the interaction between global and local features, enabling the model to build a more holistic understanding of the spatial relationships within the brain.
(3) Suppression of Irrelevant Information and Improved Generalization: MRI images contain substantial irrelevant information, including cerebrospinal fluid, blood vessels, and scanning artifacts, which can interfere with traditional CNN-based feature extraction. Spatial attention mechanisms help suppress such background interference, enhancing the model’s ability to focus on AD-relevant features and improving its generalization capability while reducing misclassification.
(4) Adaptability to Transformer Architectures for Long-Range Dependency Modeling: Traditional CNNs are limited by their local receptive fields and struggle with modeling distant spatial dependencies. Integrating spatial attention mechanisms within architectures such as the Swin Transformer enables modeling of cross-regional spatial correlations. This enhances the model’s capacity to capture long-range dependencies and improves the overall quality of feature representation.

### Limitations of existing models and research motivation

Given the feature extraction requirements for AD MRI images outlined above, current mainstream deep learning models face the following specific and complementary challenges when applied to this task, which directly hinder their ability to identify key pathological features such as hippocampal atrophy:

Limitations of CNN Models: Lack of Long-range Dependency Modeling. While CNNs excel at extracting local features (e.g., edges, textures), their inherent design based on local receptive fields makes it difficult to effectively model long-range spatial dependencies across brain regions (Chen et al., 2021). In the context of AD diagnosis, this implies that a CNN might “see” hippocampal atrophy in isolation but struggle to correlate it with atrophy patterns in distant regions (e.g., cortex associated with the default mode network). Such global correlation is crucial for distinguishing AD from other types of dementia.

Limitations of Standard ViT Models: Computational Inefficiency and Insufficient Local Sensitivity. The global self-attention mechanism of Vision Transformers (ViTs) can theoretically capture dependencies across the entire image. However, its computational complexity scales quadratically with image resolution, leading to high costs for processing high-resolution MRI. More critically, its uniform attention computation over all image patches may dilute the focus on subtle, localized pathological changes critical for AD (e.g., early entorhinal cortical thinning). The model may treat brain regions containing lesions and uninformative background with equal importance, failing to leverage the prior knowledge of spatial locality of lesions in medical images.

Limitations of Swin Transformer: Delayed Acquisition of Global Context Across Windows. Swin Transformer achieves an excellent trade-off between efficiency and modeling power through its local window and shifted window mechanisms (Liu et al., 2021). However, its core attention computation is confined within non-overlapping local windows. Although hierarchical design and window shifting facilitate cross-window information exchange, this interaction is indirect and involves latency. In the early stages of the network (e.g., Stage 1), the model lacks an immediate, explicit mechanism to inject global contextual information from the image’s rows and columns (vertical/horizontal axes) into the features within each window. This may limit the model’s ability to quickly establish associations between AD-relevant features distributed across different windows (e.g., the hippocampus and signs of ventricular enlargement located on different image rows) at an early layer.

Therefore, the core technical problem addressed in this study is: How to design a lightweight plug-and-play module for efficient vision architectures, exemplified by Swin Transformer, to compensate for their potential deficiency in immediately modeling global spatial context (especially along anatomical axes) in the early network stages, thereby guiding the model to focus more precisely and robustly on AD-related anisotropic pathological features.

Our proposed Bidirectional Spatial Attention (ATT) module is designed specifically to address the above issues. By performing adaptive pooling and feature interaction separately along the height and width dimensions, it explicitly encodes the global statistical information of each spatial position’s row and column with linear complexity. Integrating it into Stage 1 of the Swin Transformer acts as a low-cost global spatial feature calibrator at the forefront of the network. This allows the model to preemptively enhance its sensitivity to variations along key anatomical axes before entering the local window-based computation.

### Structural design of the ATT mechanism

In this study, the custom attention module (ATT) is chosen over traditional channel attention or global attention mechanisms for two main reasons. On one hand, the changes in Alzheimer’s disease (AD) MRI images are typically manifested as complex spatial features (such as brain region atrophy or lesions). Compared to channel attention mechanisms (Guo et al., 2022), the custom ATT module not only optimizes feature representation at the channel level but also considers both local and global information in the image, thus overcoming the limitations of channel attention in modeling spatial information. On the other hand, not all regions in AD MRI images require global attention, and excessive global attention may distract the network’s focus, affecting its sensitivity to key local areas. Compared to global attention mechanisms (Liu et al., 2021), the custom ATT module can focus on feature extraction in critical regions of the MRI images while flexibly adjusting the fusion of local and global information, avoiding the computational redundancy that global attention may introduce. This is particularly important for capturing subtle early-stage changes in Alzheimer’s disease.

**Figure 1:**
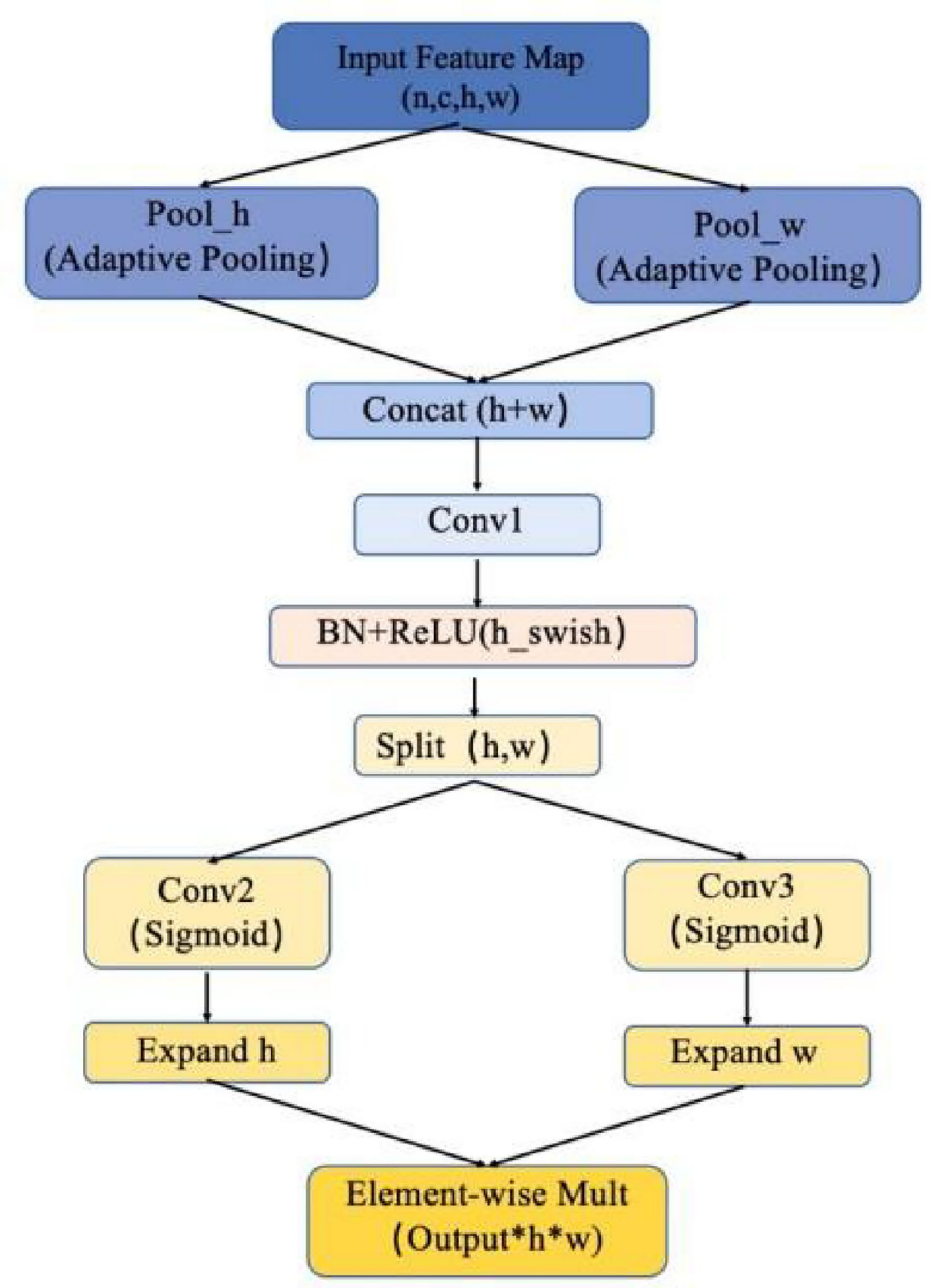
Structure diagram of the custom ATT module.

The structure of the ATT module is shown in the Figure 1, consisting primarily of adaptive pooling layers, convolutional layers, batch normalization layers, and activation functions. In the ATT module, the input feature map $x\in {\mathbb{R}}^{n\times c\times h\times w}$ first undergoes height pooling Poolh(x) and width pooling Poolw(x), both using adaptive average pooling. Specifically, the height pooling and width pooling are defined as Equations 1, 2:


$$x_{h}(i,j)=\frac{1}{W}\sum_{k=1}^{w}x(i,j,k)$$ (1)

$$x_{w}(i,j)=\frac{1}{h}\sum_{k=1}^{w}x(i,j,k)$$ (2)

where *x_h_* and *x_w_* represent the pooling results along the height and width directions, respectively. These pooling operations effectively extract spatial information, helping the model to focus on features across different spatial dimensions.

Next, *x_h_* and *x_w_* are concatenated to form a new feature map, which is then passed through a convolutional layer Conv1 for dimensionality reduction. After batch normalization and processing with the h-swish activation function, the resulting feature map is split into height and width channels. The h-swish activation function, an improved version of the Swish function, is defined as Equations 3, 4:


$$h_{\text{swish}}(x)=x\times h_{\text{sigmoid}}(x)$$ (3)

$$h_{\text{sigmoid}}(x)=\max(0,\min(1,\frac{x+3}{6}))$$ (4)

where ReLU6(*x*) = min(max(0,x),6). This activation function retains non-linearity while reducing computational complexity.

Then, independent convolution operations are applied to the height and width branches (i.e., Conv2 and Conv3), followed by the Sigmoid function to obtain height attention weights *x*_h_^′′^ and width attention weights *x*_w_^′′^. These weights are then expanded to match the shape of the input feature map and applied to it through element-wise multiplication. Specifically, each element of the output feature map youtputyoutput is computed as Equation 5:


$$y_{\text{output}}(i,j)=x(i,j)\cdot xh^{\prime \prime }(i)\cdot xw^{\prime \prime }(j)$$ (5)

This element-wise multiplication mechanism applies the height and width attention weights across the spatial dimensions through broadcasting, effectively enhancing prominent features in the input feature map and improving the model’s non-linear representational capacity. ATT module structure diagram is shown in Figure 1.

When data flows through this module, an input tensor of shape (n,c,h,w) is first received, where n represents the batch size, c indicates the number of channels, and h and w denote the height and width, respectively. The processing flow of the ATT module can be summarized in the following steps:

Step 1: The input tensor undergoes adaptive average pooling along the height and width dimensions. The pooling operation along the height dimension is performed by self.pool_h, producing an output tensor with shape (n,c,h,1), thereby retaining the height while compressing the width to 1. Similarly, the pooling operation along the width dimension is executed using self.pool_w and the dimensions are rearranged through a permute operation to generate a tensor of shape (n,c,w,1). The pooled results from both dimensions are concatenated along the height dimension to form a new tensor of shape (n,c,h + w,1). This step ensures that the module captures the most salient features across different scales, even when the brain atrophy varies across patients or regions. By compressing spatial dimensions and focusing on relevant features, the network becomes less sensitive to irrelevant information and more attuned to the characteristic structural changes in the brain caused by Alzheimer’s disease.
Step 2: The concatenated tensor is then processed by a convolutional layer to reduce its dimensionality. This convolutional layer serves to decrease the number of channels (by adjusting to the parameter mip) and extract more compact features. Subsequently, the output tensor is passed through a batch normalization layer followed by the activation function h_swish to ensure data stability and enhance the model’s nonlinear expressiveness. At this stage, the output tensor is split into height and width branches, corresponding to shapes (n,mip,h,1) and (n,mip,w,1), respectively. Each branch is further processed through independent convolutional operations to generate the corresponding attention weights. The height branch, after convolution, utilizes a sigmoid activation function to produce attention weights of shape (n,oup,h,1); similarly, the width branch yields attention weights of shape (n,oup,1,w). To integrate these weights with the original input, they are expanded along the height and width dimensions to match the input shape, resulting in shapes of (n,oup,h,w). This ensures that only the most essential features are retained, making the subsequent attention mechanism more effective.
Step 3: The ATT module achieves bidirectional spatial attention fusion by multiplying the original input with the generated height and width attention weights. Specifically, the initial input tensor is element-wise multiplied by the attention weights along both the height and width dimensions, yielding the final output. This process ensures that the model can simultaneously focus on important spatial features across both dimensions, significantly enhancing the feature representation capability of the image. In Alzheimer’s MRI scans, the spatial relationships between different brain regions are crucial for identifying pathological changes. The bidirectional attention mechanism allows the model to focus not only on specific regions within the height dimension (e.g., specific brain slices or sections) but also on the width dimension (e.g., voxel relationships across different anatomical structures). By integrating attention weights from both spatial directions, the model can capture the interactions between various regions of the brain, leading to a more robust understanding of the disease’s progression.

The ATT module enhances the model’s flexibility in capturing critical information within images, particularly in handling complex spatial structures, thereby significantly improving the model’s expressiveness. This module design strengthens the model’s understanding of spatial features while maintaining computational efficiency, making it a vital component for enhancing performance in image classification or detection tasks.

### Innovative model

We propose the Swin-Tiny+ATT model based on the Swin-Transformer architecture. Given that the research task does not require processing highly complex inputs (the MRI images in the OASIS1 dataset are primarily T1-weighted structural images, with a resolution typically ranging from 1mm^3^ to 1.5mm^3^, and spatial dimensions of 256 × 256), the MRI images in the OASIS1 dataset can be considered as relatively low resolution for the Swin Transformer model. Swin-Tiny is sufficient to meet the requirements of the task. For mid-sized datasets, using larger variants such as Swin-Transformer-Base or Swin-Transformer-Large may lead to overfitting without providing significant improvements in performance (Jiang et al., 2025). In addition, we conducted resource consumption tests for other variants, using Max Memory Used as the metric. The specific details are shown in Table 1. Therefore, we have chosen Swin-Tiny over other versions as the base model.

**Table 1:** The number of parameters and memory usage of different Swin Transformer variants.

Variants of Swin Transformer	Number of parameters	Max memory used
Swin -Tiny	28 M	429.82 MiB
Swin -Small	50 M	763.20 MiB
Swin -Base	88 M	1437.15 MiB
Swin -Large	197 M	3416.16MiB

The hierarchical design of Swin Transformer emphasizes the distinct roles of different layers in information processing and feature abstraction. The ATT module is primarily used to capture long-range dependencies and is typically placed in layers that effectively capture global information. Introducing the ATT module in the first Swin block helps capture low-level features and enhance local information flow, particularly in image processing tasks, where it is effective in capturing details like edges and textures. As the network deepens, later layers rely more on local information aggregation, and using the ATT module may introduce excessive computational complexity and the risk of overfitting. Therefore, we only apply the ATT module in stage 1 of our model, which improves performance while maintaining computational efficiency and preventing overfitting, striking a balance between performance and computational cost.

**Figure 2:**
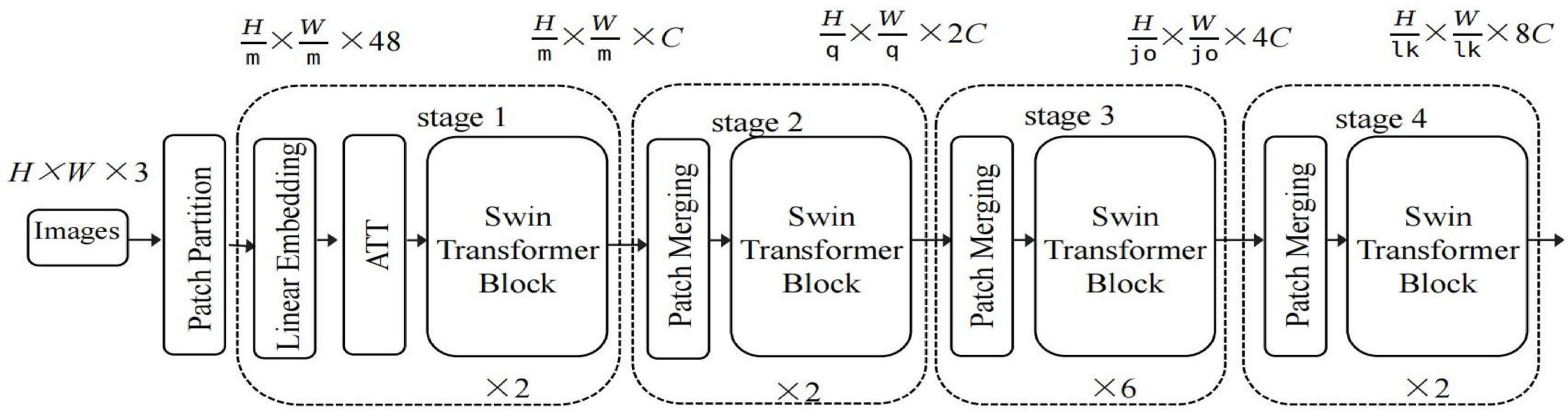
Structure diagram of the Swin Transformer Network with ATT. The network structure illustrates the dual-path spatial attention mechanism designed for enhancing the feature representation of medical images. The input feature map undergoes height-wise and width-wise adaptive average pooling separately to capture global spatial dependencies. These pooled features are then concatenated, processed through a convolutional layer for dimensionality reduction, activated by the h-swish function, and split into independent height and width branches. Each branch generates attention weights via convolution and a sigmoid function, which are subsequently applied to the original input through element-wise multiplication. This bidirectional weighting scheme enables the module to emphasize informative regions along both spatial dimensions, thereby improving the model’s ability to focus on clinically relevant structural patterns in MRI scans.

The structure of the Swin Transformer network with the added custom attention module (ATT) is illustrated in Figure 2.

The algorithm flow of this study is shown in Table 2.

**Table 2:** Network algorithm flow of Swin-Tiny+ ATT.

Steps	Details
1. Image preprocessing	The initial image feature extraction is performed, dividing the image into multiple 4 × 4 patches, each containing local features.
2. Feature embedding	The image patches are projected into a 96-dimensional embedding space, transforming the feature map shape from (B,3,224,224) to (B,96,56,56), where B represents the batch size.
3. Bidirectional spatial attention module (ATT)	The attention weights are generated using bidirectional pooling and convolution, which are fused with the input features to enhance spatial feature representation. The output feature map retains the shape (B,96,56,56).
4. Flattening and transforming	The feature map is flattened and reshaped to (B,3136,96), preparing it for the subsequent window attention mechanism.
5. Feature extraction (Swin Transformer Block)	The feature extraction process includes normalization, window partitioning and shifting, window attention mechanism, window reversal, residual connection, and multi-layer perceptron (MLP) for final output feature extraction. Detailed steps are as follows:
(5.1) Normalization	Layer normalization is applied to the flattened feature map to ensure balanced data distribution.
(5.2) Window partitioning and shifting	The feature map is divided into multiple windows, and window independence is mitigated by a shift operation, enhancing global feature interaction.
(5.3) Window attention	The multi-head attention mechanism is employed to enhance local feature representation within each window.
(5.4) Window reverse	The features after window attention processing are restored to their original shape in order to share information with subsequent network layers.
(5.5) Residual connection and MLP	Residual connections ensure that the original input information is retained, while higher-dimensional features are extracted and linearly mapped using LayerNorm and MLP components.
(5.6) Output	After multiple layers of feature extraction and residual operations, the output features are forwarded to the next module.
6. Dimensionality reduction	At the end of each stage, patch merging is performed to reduce the size of the feature map and lower computational complexity. This process involves convolution and pooling operations.
7. Classification head	Global average pooling is used to convert the feature map into fixed-length vectors, which are subsequently classified by a fully connected layer.

To investigate the impact of ATT module placement within the network, we conducted an ablation study by inserting the module into different stages of Swin-Tiny (Stage 1 to Stage 4). Additionally, to assess computational trade-offs, we measured memory usage and inference time for each configuration. The goal was to identify the optimal balance between performance enhancement and resource overhead.

## Results

### Experimental settings

The experiments in this study are based on the PyTorch framework, implemented in Python with Python 3.8 on a PyCharm Community 2023.3.4 integrated development environment, running on a Linux operating system with four NVIDIA Tesla V100 GPUs. The server model, C4140, includes a 32-core CPU, 512GB of RAM, and a 6 TB storage capacity, while each GPU has 32GB of memory. The network input size is set to 224 × 224. During model training, the initial learning rate is set to 1e-2, optimized using the Stochastic Gradient Descent (SGD) algorithm, with a momentum parameter of 0.95 and a cosine learning rate decay. Compared to adaptive optimizers such as Adam, SGD is more effective in preventing overfitting in computer vision tasks, making it particularly suitable for deep learning models like Swin Transformer. We adopt the cosine learning rate decay strategy, which gradually reduces the learning rate, allowing the model to transition smoothly from rapid exploration in the early training stages to fine-tuned optimization in later stages. This approach enhances training stability and improves overall performance. Weight decay is set to 1e-3, and the cross-entropy loss function is applied. We employ the cross-entropy loss function (Gonzalez and Miikkulainen, 2020), which is well-suited for this classification task as it effectively measures the discrepancy between the predicted probability distribution and the true class labels. Cross-entropy loss encourages the model to produce more discriminative probability distributions. Additionally, this loss function naturally aligns with the Softmax activation function, helping the model generate normalized probability distributions and improving classification reliability. The batch size is configured as 64, and the model is trained for 300 epochs.

To rigorously evaluate the statistical significance of the performance improvement, we conducted 20 independent experiments with different random seeds for data splitting and model initialization. For each experiment, the dataset was randomly partitioned into training, validation, and test sets according to the 8:1:1 ratio. Both the baseline Swin-Tiny model and our proposed Swin-Tiny+ATT model were trained and evaluated under identical conditions in each run. The performance difference between the two models was then assessed using a paired *t*-test on the results from these 20 runs. Additionally, the 95% confidence interval for the mean accuracy improvement was calculated.

### Experimental DATAS

#### Dataset

The study employs the OASIS (Open Access Series of Imaging Studies) OASIS-1 dataset (Marcus et al., 2007), which comprises cross-sectional MRI data of young adults, middle-aged, non-demented, and demented elderly individuals. This dataset includes 416 subjects aged 18 to 96, each with 3 or 4 separate T1-weighted MRI scans. Among these, 100 elderly individuals aged over 60 were diagnosed with either mild or moderate Alzheimer’s Disease (AD), alongside 20 uncorrected participants whose data were collected during subsequent follow-ups. The dataset is available at http://www.oasis-brains.org/. Within the OASIS-1 dataset, MRI images are classified based on Alzheimer’s severity into four categories:

- NonDemented: Representing subjects with no dementia diagnosis, typically used as the control group.
- VeryMildDemented: Representing subjects with very mild Alzheimer’s Disease.
- MildDemented: Representing subjects with mild Alzheimer’s Disease.
- ModerateDemented: Representing subjects with moderate Alzheimer’s Disease.

In this study, the VeryMildDemented category is incorporated into the MildDemented category. From a neuroimaging perspective, there are no significant differences in cortical atrophy or hippocampal volume reduction between patients in the Very Mild Dementia and Mild Dementia stages, especially in the early stages of Alzheimer’s disease. Furthermore, distinguishing between these two categories in neuroimaging analysis is often not of decisive significance. In clinical practice, there is no clear boundary between the Very Mild Dementia and Mild Dementia stages in the early stages of Alzheimer’s disease, as the delineation between these two stages is often ambiguous due to individual differences (Gonzalez and Miikkulainen, 2020). The assessment of cognitive impairment is highly subjective. Therefore, combining these two categories can simplify neuroimaging analysis, enhance the stability and effectiveness of statistical analyses, and reduce the impact of such subjectivity. All data is anonymized to comply with public distribution requirements. The dataset is split into training, testing, and validation sets at an 8:1:1 ratio, with details shown in Table 3. Additionally, images in the ModerateDemented category in the training set are augmented by a factor of 50. The data splitting was performed at the patient level, ensuring that images from the same subject do not appear across the training, validation, and test sets.

**Table 3:** Dataset allocation details.

Dataset	NonDemented	MildDemented	ModerateDemented
Training sets	2,560	2,509	52*50
Testing sets	320	313	6
Validation sets	320	313	6
The three MRI categories are shown as follows	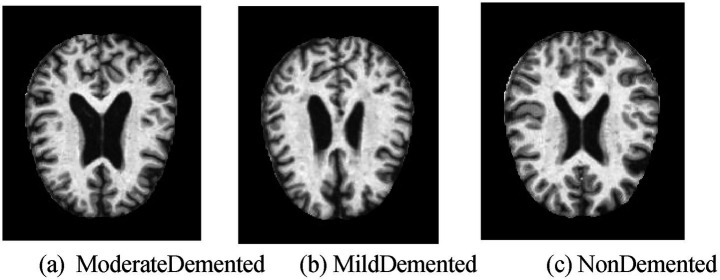		

#### Data preprocessing

In order to ensure the consistency and quality of image inputs, we performed both normalization and standardization on the MRI images. First, the images were normalized by dividing the pixel values by 255, scaling them to the range of [0, 1], which helps mitigate the impact of different scanning devices, patients, or scanning parameters. Subsequently, the images underwent standardization by subtracting the mean ([0.485, 0.456, 0.406]) and dividing by the standard deviation ([0.229, 0.224, 0.225]) based on the ImageNet dataset. This step adjusts the pixel intensity distribution across the channels, eliminating intensity differences between images. Through this preprocessing procedure, all input images were scaled to a consistent range, improving the efficiency and performance of model training.

In the data preprocessing phase, data augmentation (Shorten and Khoshgoftaar, 2019) and balance (Zhang et al., 2021) are applied to increase the quantity and variability of the dataset. Initially, images are converted to RGB format to ensure consistent color formatting across input images. Data augmentation, a critical component in deep learning image classification tasks, is then performed to enhance the model’s generalization and robustness (Zamir et al., 2020). In this study, random and automated augmentations are utilized, incorporating a range of image processing techniques, including geometric transformations and color space adjustments (Shorten and Khoshgoftaar, 2019).

During the data augmentation phase, we implemented a tailored strategy designed specifically for the characteristics of structural brain MRI in Alzheimer’s Disease. This strategy encompasses two main categories of operations, the selection and parameterization of which are grounded in medical rationale:

Geometric Transformations: These include random translation, rotation, and shearing. These operations are designed to simulate common anatomical variations across individuals (e.g., head position, angle) and alignment differences between scanners encountered in clinical practice. Critical parameters are strictly constrained (e.g., rotation is limited to ±30 degrees) to ensure that augmented images preserve anatomical plausibility, preventing key diagnostic regions (e.g., the hippocampus) from being displaced out of view or severely distorted due to excessive transformation. This guides the model to learn morphological features that are invariant to position and orientation.

Intensity/Color Space Transformations: These include adjustments to contrast, brightness, and operations such as “Solarize.” These transformations are not intended to alter color but to simulate the variations in signal intensity and contrast that may arise in MRI images due to different devices and acquisition protocols. By introducing this controlled intensity heterogeneity, we significantly enhance the model’s generalization capability and robustness to multi-center, multi-scanner data.

All augmentation operations are applied in stochastically combined sequences to maximize the diversity of generated samples. We deliberately avoided augmentation methods that could randomly erase critical small-scale biomarkers (e.g., “Random Erasing” or “Cutout”), ensuring that all training samples retain complete pathological information. The effectiveness of this customized strategy has been validated through ablation studies (see Table 4), confirming its crucial and positive contribution to model performance improvement.

**Table 4:** Performance comparison of different models with and without ATT module (Mean ± Std over 20 runs).

Model family	Model	ATT module	Accuracy	Recall	Precision	F1-score
CNN baselines	VGG16bn	No	81.42% ± 0.18%	79.24% ± 0.22%	80.01% ± 0.20%	79.19% ± 0.19%
	VGG16bn	Yes	82.10% ± 0.15%	80.28% ± 0.19%	81.39% ± 0.17%	80.83% ± 0.16%
	MobileNetV2	No	79.15% ± 0.25%	77.82% ± 0.28%	76.30% ± 0.30%	77.05% ± 0.26%
	MobileNetV2	Yes	80.00% ± 0.22%	78.87% ± 0.25%	78.45% ± 0.27%	78.66% ± 0.23%
Vision transformers	ViT_b_16	No	73.84% ± 0.35%	68.79% ± 0.40%	75.34% ± 0.32%	71.43% ± 0.36%
	ViT_b_16	Yes	75.07% ± 0.30%	70.51% ± 0.35%	76.83% ± 0.28%	73.13% ± 0.31%
	MaxViT-T	No	86.05% ± 0.15%	87.45% ± 0.18%	86.75% ± 0.17%	87.10% ± 0.16%
	MaxViT-T	Yes	87.25% ± 0.13%	88.95% ± 0.16%	88.15% ± 0.15%	88.55% ± 0.14%
	BEiT-3	No	85.07% ± 0.20%	86.73% ± 0.22%	84.54% ± 0.23%	85.62% ± 0.21%
	BEiT-3	Yes	85.93% ± 0.18%	87.07% ± 0.20%	85.85% ± 0.21%	86.45% ± 0.19%
	Swin-Small	No	84.01% ± 0.23%	83.81% ± 0.27%	89.35% ± 0.25%	86.26% ± 0.24%
	Swin-Base	No	67.37% ± 0.35%	55.39% ± 0.40%	55.09% ± 0.38%	55.24% ± 0.36%
Our architecture	Swin-Tiny	No	84.79% ± 0.21%	89.77% ± 0.25%	85.22% ± 0.23%	87.21% ± 0.22%
	Swin-Tiny+ATT	Yes	87.91% ± 0.12% (95% CI:87.85–87.97%)	91.87% ± 0.15%	91.93% ± 0.14%	91.84% ± 0.13%

Data augmentation increases the diversity of the training data through geometric transformations and color space adjustments. These transformations help the model learn features from different perspectives and scales, thereby improving its ability to adapt to unseen data. The augmented data enables the model to generalize better to real-world scenarios, avoid overfitting, and enhance its performance under various environments and conditions.

To address the data imbalance in the OASIS-1 dataset, specifically for the underrepresented ModerateDemented category, each MR image in this category is augmented to generate 50 additional training samples per image. This expansion results in a balanced training dataset where the class ratio for NonDemented, MildDemented, and ModerateDemented categories approximates 1:1:1. This helps mitigate the negative impact of the scarcity of samples from any single category. However, if the augmentation factor is too large (e.g., by overly duplicating samples from the minority class), it may lead to overfitting. Therefore, when augmenting the sample size, we carefully considered the diversity of the augmentation strategies. Instead of simply replicating the original samples of the minority class, we apply various data augmentation techniques to generate new samples. These augmented samples introduce different perspectives and details by transforming the original data, thus increasing the diversity and variability of the data and preventing the model from overfitting to specific samples. To further avoid overfitting, we also applied regularization techniques, such as dropout and L2 regularization, during training.

**Figure 3:**
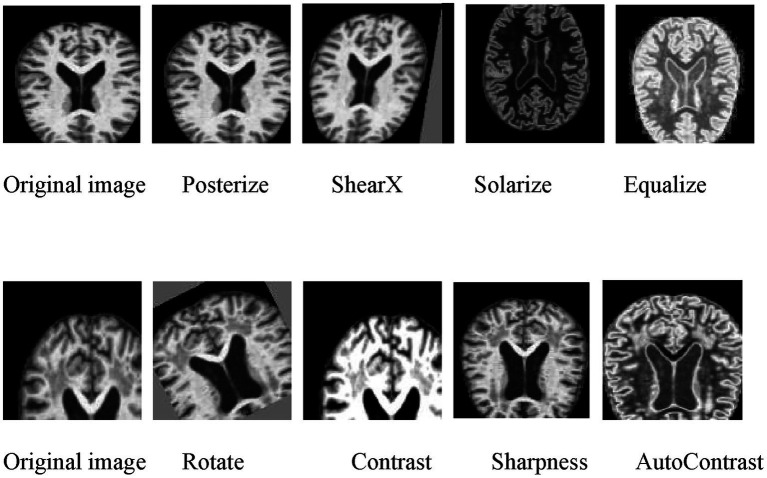
Image after data augmentation.

Below are samples illustrating the results after data preprocessing (Figure 3).

### Evaluation metrics

The model’s performance is evaluated using standard classification metrics, including Accuracy, Precision, Recall, and the F1 Score (Grandini et al., 2020).

Accuracy (Acc): Accuracy represents the proportion of samples correctly classified by the model out of the total sample size, with higher accuracy indicating a stronger classification capability. The calculation formula is as Equation 6:


$$\mathrm{Acc}=\frac{\mathrm{TP}+\mathrm{TN}}{\mathrm{TP}+\mathrm{TN}+\mathrm{FP}+\mathrm{FN}}$$ (6)

Precision (Pre): Precision indicates the proportion of true positive samples among all samples predicted as positive by the model. In other words, it measures the accuracy of the model’s positive predictions. The formula for precision is as Equation 7:


$$\mathrm{Pre}=\frac{\mathrm{TP}}{\mathrm{TP}+\mathrm{FP}}$$ (7)

Recall (Rec): Recall represents the proportion of true positive samples among all actual positive samples, thereby assessing the model’s ability to identify positive samples. The calculation formula for recall is as Equation 8:


$$\mathrm{Rec}=\frac{\mathrm{TP}}{\mathrm{TP}+\mathrm{FN}}$$ (8)

F1 Score: The F1 Score is the harmonic mean of Precision and Recall, reflecting both the accuracy and recognition capability of the model. Ranging between 0 and 1, higher F1 values indicate better model performance. The F1 Score is calculated as Equation 9:


$$F_{1}=2\cdot \frac{\mathrm{Pre}\times \mathrm{Rec}}{\mathrm{Pre}+\mathrm{Rec}}$$ (9)

Where TP (True Positive) represents the number of positive samples correctly classified by the model, TN (True Negative) is the count of negative samples correctly classified, FP (False Positive) represents negative samples incorrectly classified as positive, and FN (False Negative) denotes positive samples incorrectly classified as negative. Here, “positive” refers to patients, while “negative” refers to healthy individuals.

### Experimental results

#### Comparison of classification results from different models

In the task of Alzheimer’s disease MR image classification, this study systematically evaluates the performance of various models, including CNNs, Vision Transformers, and their advanced variants under identical dataset and experimental platform conditions, and verifies the generalizability of the proposed ATT module. The experimental results are shown in Table 4.

The performance of different models varies significantly in this task. The Swin-Tiny+ATT model achieves the best performance, with a mean accuracy of 87.91% ± 0.12%, notably outperforming CNN baselines such as VGG16bn (81.42% ± 0.18%) and MobileNetV2 (79.15% ± 0.25%), as well as the original ViT_b_16 (73.84% ± 0.35%) and advanced models including MaxViT-T (86.05% ± 0.15%) and BEiT-3 (85.07% ± 0.20%).

More importantly, the ATT module demonstrates strong cross-architecture generalization capability. When integrated into VGG16bn, MobileNetV2, ViT_b_16, MaxViT-T, and BEiT-3, all performance metrics show consistent and measurable improvement (see Table 3), validating its effectiveness as a general-purpose feature enhancement component. However, the ATT module yields the most significant performance gain within the Swin-Tiny architecture, highlighting the synergistic design advantages between the two.

Compared to the baseline Swin-Tiny model without the ATT mechanism (mean accuracy 84.79% ± 0.21%), the improvement with Swin-Tiny+ATT is statistically significant. Based on 20 independent runs, a paired t-test on accuracy values confirms a highly significant difference (t(19) = 55.37, *p* < 0.0001), providing strong evidence against the null hypothesis. The 95% confidence interval for the mean accuracy improvement is [3.02, 3.22%].

Furthermore, this study evaluates the performance of Swin Transformer variants of different scales. The results show that Swin-Tiny and Swin-Small perform better in terms of accuracy, recall, and other metrics. After integrating the ATT mechanism, their performance is significantly improved. However, as the model size increases to Swin-Base, the performance metrics decline noticeably, indicating that larger models not only substantially increase computational resource consumption but may also lead to diminishing returns in performance due to overfitting or increased optimization difficulty.

In summary, the Swin-Tiny+ATT model achieves the best performance in this task. Extensive cross-model experiments demonstrate that the ATT module is an effective general enhancement component, while its combination with the hierarchical window attention mechanism of Swin Transformer constitutes an efficient solution for modeling the complex spatial features of Alzheimer’s disease MRI images.

**Figure 4:**
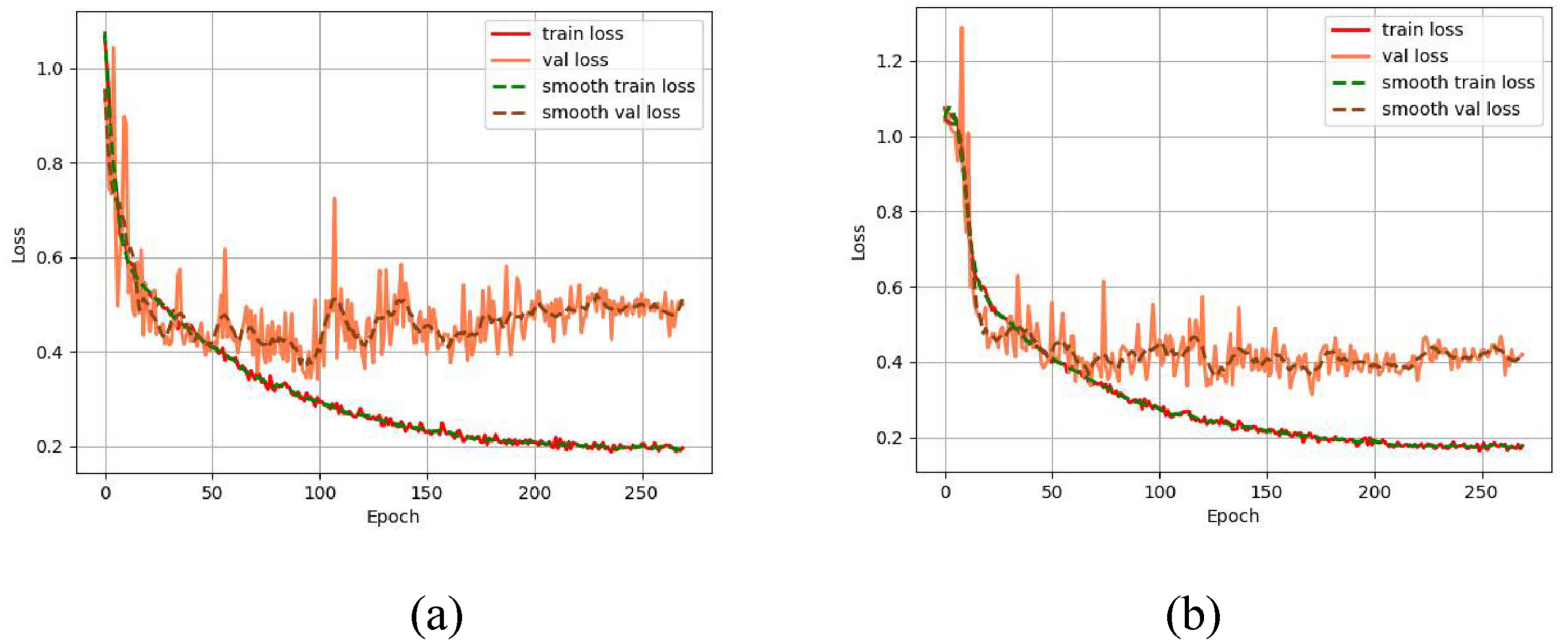
**(a)** Loss curve of Swin Transformer. **(b)** Loss curve of Swin-Tiny+ATT.

The loss curves of the baseline Swin Transformer model and the improved Swin-Tiny+ATT model are shown in the figure: From the comparison of the loss curves in Figures 4a,b, it can be observed that after incorporating the ATT mechanism, both the training loss and validation loss of the Swin Transformer model decrease more smoothly, and the final validation loss is lower. Notably, in the early stages of training, the model with the ATT mechanism significantly reduces the fluctuation in loss, validating the effectiveness of the ATT mechanism in improving model stability and generalization performance. Overall, the Swin-Tiny+ATT model exhibits improvements in both convergence speed and final performance compared to the original model.

To more accurately evaluate the Alzheimer’s disease MR image classification model, the confusion matrices of the baseline model Swin-Tiny and the Swin-Tiny+ATT model are presented. Figure 4a illustrates the confusion matrix of the baseline Swin Transformer model. This model achieved 89% accuracy in identifying the MildDemented category, 81% accuracy in the NonDemented category, and demonstrated strong performance in the ModerateDemented category with an accuracy of 100%. However, the model exhibited notable confusion, with misclassification rates of 19 and 11% in the NonDemented and MildDemented categories, respectively.

The classification results of the model for each type of MR image were tested, and the precision, recall, and F1-score were obtained, as shown in Table 5. The Swin Transformer-ATT model demonstrated significant improvements in the Alzheimer’s disease MR image classification task compared to the Swin Transformer model, especially in terms of precision, recall, and F1-score.

**Table 5:** Classification results for MRI images in each category.

Model	Metrics	NonDemented	MildDemented	ModerateDemented*
Swin-Tiny	Accuracy	88%	82%	86%
	Recall	81%	89%	100%
	F1-score	84%	85%	92%
Swin-Tiny+ATT	Accuracy	90%	86%	100%
	Recall	85%	91%	100%
	F1-score	88%	88%	100%

*The test set for the ModerateDemented class contains only 6 samples. The high accuracy should be interpreted with caution due to the limited sample size.

In the NonDemented category, the precision increased from 88 to 90%, recall improved from 81 to 85%, and the F1-score rose from 84 to 88%, representing increases of 2, 4, and 4%, respectively. In the MildDemented category, both precision and F1-score improved by approximately 4%. This indicates that the Swin Transformer-ATT model exhibits stronger generalization ability and classification performance. The improvements in recall and F1-score demonstrate the model’s enhanced accuracy in capturing the features of different dementia categories. The incorporation of the ATT mechanism has optimized the model’s ability to recognize and classify Alzheimer’s disease patients.

#### Ablation study on ATT module placement

To investigate the optimal placement strategy of the proposed ATT module within the network hierarchy, we conducted an ablation study by inserting it into different stages (Stage 1 to Stage 4) of the Swin-Tiny backbone. The performance and corresponding computational costs are summarized in Table 5. The results indicate that inserting the ATT module only in Stage 1 yields the best performance (87.96% accuracy, 91.89% F1-score) with the minimal increase in memory overhead (430.46 MiB). As the ATT module is introduced into deeper stages (e.g., Stage 1 + 2, Stage 1 + 2 + 3, and all stages), a clear trend of diminishing returns is observed: while the performance remains superior to the baseline Swin-Tiny without ATT (84.83% accuracy), it gradually decreases. Concurrently, the memory usage increases linearly with the number of stages containing the ATT module. More details are as Table 6.

**Table 6:** Performance and resource impact of ATT module at different stages in Swin-Tiny.

ATT placement	Accuracy	Recall	Precision	F1-score	Memory usage (MiB)
Stage 1 only	87.96%	91.92%	91.98%	91.89%	430.46
Stage 1+2	87.50%	91.20%	91.50%	91.35%	445.2
Stage 1+2+3	86.80%	90.50%	90.80%	90.65%	462.8
All Stages (1–4)	86.10%	89.80%	90.20%	90.00%	480.15

This phenomenon can be attributed to the distinct roles of different network depths. The early stage (Stage 1) primarily processes low-level features and basic spatial structures. Introducing the ATT module at this point effectively acts as a global spatial feature calibrator, guiding the subsequent window-based attention mechanisms to focus on anatomically relevant regions from the outset. In contrast, deeper stages are responsible for aggregating and refining high-level semantic features. Adding the ATT module to these layers may introduce redundant feature recalibration and potentially interfere with the local feature aggregation process, leading to suboptimal performance. Therefore, placing the ATT module solely in Stage 1 represents the optimal trade-off, achieving significant performance enhancement while maintaining computational efficiency. This placement strategy is adopted in our final Swin-Tiny+ATT model.

#### Effect of data augmentation

Data augmentation and expansion significantly improved model performance in this study, particularly in addressing issues of data scarcity and class imbalance. By introducing diverse training data, the accuracy of the Swin Transformer model increased from 70.52 to 84.83%, effectively preventing overfitting and enhancing the model’s generalization ability on test data. Additionally, data augmentation effectively mitigated the issue of sample imbalance in the OASIS1 dataset, with the recall of the Swin Transformer model rising from 47.39 to 89.82%, significantly improving the model’s ability to recognize minority class samples.

Moreover, data augmentation enhanced the model’s capacity to handle complex MR image features, with the F1 score of the MobileNetV2 model increasing from 76.26 to 83.70%. By applying various geometric and color space transformations, the model was better equipped to handle images under different viewpoints, lighting conditions, and contrast levels, enabling more accurate identification of Alzheimer’s disease-related features.

Lastly, data augmentation and expansion significantly boosted the robustness of the models. The F1 scores of the VGG16bn and Swin Transformer models improved from 50.23 and 47.00% to 79.23 and 87.26%, respectively, demonstrating that the models maintained strong performance even with complex and varied data. Therefore, data augmentation and expansion effectively addressed challenges such as data insufficiency, class imbalance, and feature complexity, significantly enhancing the overall performance of Alzheimer’s disease MR image classification models. The data augmentation effect is shown in Table 7.

**Table 7:** Comparison of model evaluation indexes before and after data augmentation.

Model	Data augmentation	Accuracy	Recall	Precision	F1-score
VGG16bn	×	75.53%	50.94%	52.61%	50.23%
VGG16bn	√	**81.47%**	**79.29%**	**80.05%**	**79.23%**
mobilenetv2	×	80.92%	79.01%	74.30%	76.26%
mobilenetv2	√	**82.72%**	**80.21%**	**88.45%**	**83.70%**
vit_b_16	×	70.60%	47.54%	47.18%	47.28%
vit_b_16	√	**73.89%**	**68.84%**	**75.39%**	**71.48%**
Swin-Tiny	×	70.52%	47.39%	47.42%	47.00%
Swin-Tiny	√	**84.83%**	**89.82%**	**85.27%**	**87.26%**
Swin-Tiny+ATT	×	73.49%	49.42%	49.12%	49.14%
Swin-Tiny+ATT	√	**87.96%**	**91.92%**	**91.98%**	**91.89%**

We present the experimental results of the model with data augmentation in bold.

## Conclusion

This study significantly enhanced the accuracy and robustness of Alzheimer’s disease (AD) MR image classification by improving the Swin Transformer model and incorporating a custom bidirectional spatial attention module (ATT). Experimental results show that the addition of the ATT module improved the performance of the Swin Transformer model, with accuracy increasing from 84.83 to 87.96%, recall from 89.82 to 91.92%, precision from 85.27 to 91.98%, and F1 score from 87.26 to 91.89%. Furthermore, the ATT module did not significantly increase memory usage, with the Max memory consumption of Swin Transformer being 429.82 MiB and Swin-Tiny+ATT being 430.46 MiB. These improvements demonstrate that the ATT module plays a crucial role in capturing complex spatial features and boosting the model’s ability to distinguish between MildDemented and NonDemented categories.

This study fully demonstrates that the proposed ATT module exhibits strong architectural versatility—it not only effectively enhances the performance of Swin Transformer but also consistently improves accuracy across various backbone networks, including VGG16bn, MobileNetV2, ViT_b_16, and even advanced models such as MaxViT and BEiT-3. This confirms its broad applicability as a lightweight, plug-and-play feature enhancement component. Meanwhile, ablation experiments reveal that placing the ATT module in the shallower layers of the network (e.g., Stage 1) achieves the best performance-efficiency trade-off, as early-stage spatial feature calibration more effectively guides subsequent layers to focus on globally relevant anatomical structures associated with pathology.

By implementing data augmentation strategies, this study effectively addressed sample insufficiency and class imbalance issues, preventing overfitting and enhancing the model’s generalization ability. Specifically, data augmentation increased the accuracy of the Swin Transformer model from 70.52 to 84.83%, recall from 47.39 to 89.82%, and F1 score from 47.00 to 87.26%, significantly improving the model’s capacity to identify minority class samples.

The proposed model not only improves classification accuracy but also offers practical applications in clinical settings. Integrating this approach into clinical workflows could assist clinicians in providing faster, more accurate diagnoses, leading to timely interventions for Alzheimer’s patients. The ability of the model to handle class imbalance and data scarcity, common challenges in medical imaging, makes it a valuable tool in real-world clinical scenarios. Furthermore, the integration of the bidirectional spatial attention module enhances the model’s ability to identify subtle variations in brain structures, crucial for accurate AD diagnosis. In practical terms, the incorporation of AI-based diagnostic tools like the proposed model could reduce the burden on radiologists and clinicians by automating routine diagnostic processes. It could be seamlessly integrated into existing MRI scanning systems, offering real-time analysis and supporting clinical decision-making.

In conclusion, this study demonstrates that the Swin-Tiny+ATT model excels in Alzheimer’s disease MR image classification, particularly in terms of accuracy, recall, and overall classification ability. The findings provide strong support for early Alzheimer’s disease diagnosis and offer promising avenues for future research in this field.

## Discussion

While the training dataset was balanced, the testing dataset exhibited significant imbalance in the ModerateDemented category, with only six samples. This imbalance could lead to unreliable performance evaluations for this category. Although the confusion matrix shows high accuracy in the ModerateDemented category, the limited sample size may cause biased performance metrics. Therefore, future research should aim to increase the sample size in the ModerateDemented category within the testing dataset to obtain more reliable performance assessments. To mitigate the overfitting risk, we suggest future research efforts focus on increasing the sample size in the testing dataset, especially for underrepresented categories, and incorporating regularization techniques or cross-validation methods. Additionally, exploring data augmentation strategies, such as adding random noise or utilizing GANs to generate synthetic data, could help improve the model’s robustness and generalizability. These steps would help ensure that the model perfor.

The Swin Transformer model has shown strong performance, and future research could explore hybrid architectures (Fu et al., 2022; Lu et al., 2023), self-supervised learning (Xie et al., 2021), few-shot learning (Hiller et al., 2022; Wu et al., 2022), and multimodal integration (Iqbal and Sharif, 2023; Cai et al., 2023) to further enhance its practical applications. Combining Swin Transformer with lightweight convolutional or Transformer models could lead to more efficient hybrid structures. To reduce dependence on large labeled datasets, future work could focus on self-supervised and few-shot learning techniques.

Additionally, exploring the application of Swin Transformer in cross-modal tasks, such as integrating text, speech, and video, could further improve its performance. While geometric and color transformations used in this study are effective, they may be vulnerable to adversarial attacks. Alternative methods, such as adding random white noise or employing Generative Adversarial Networks (GANs) to synthesize images (Xie et al., 2021; Creswell et al., 2018), could further strengthen model robustness. Future research should also explore additional data augmentation techniques, which would contribute to more efficient early detection and intervention for Alzheimer’s disease, ultimately improving patients’ quality of life (Iqbal and Ali, 2018).

A key limitation of this study lies in the small sample size of the “ModerateDemented” category in the test set (only 6 cases, as shown in Table 3). This undermines the statistical power of the perfect 100% metrics reported for this category in Table 4 and raises concerns about potential overfitting, making it difficult to reliably assess the model’s true generalizability for this class. To directly address this issue and rigorously validate the proposed method, our immediate future work includes: (1) evaluating the model on larger, independent external datasets (e.g., ADNI) to test its clinical generalizability across different centers and scanners; and (2) applying the proposed ATT module to other medical imaging tasks (e.g., chest X-ray classification) to verify its effectiveness as a general architectural improvement for cross-domain applicability.

## Data Availability

The original contributions presented in the study are included in the article/supplementary material, further inquiries can be directed to the corresponding author.
